# HIV-1 molecular transmission network and drug resistance in Chongqing, China, among men who have sex with men (2018–2021)

**DOI:** 10.1186/s12985-023-02112-0

**Published:** 2023-07-13

**Authors:** Tianyu Tan, Chongyang Bai, Rongrong Lu, Fangfang Chen, Long Li, Chao Zhou, Xu Xiang, Wei Zhang, Ling Ouyang, Jing Xu, Houlin Tang, Guohui Wu

**Affiliations:** 1 Chongqing Center for Disease Control and Prevention, 400042 Chongqing, China; 2 National Center for AIDS/STD Control and Prevention, Chinese Center for Disease Control and Prevention, Beijing, 102206 China

**Keywords:** Chongqing, MSM, HIV-1 transmission network, Drug resistance

## Abstract

**Background:**

Over the past few years, HIV transmission among men who have sex with men (MSM) in China has increased significantly. Chongqing, located in the southwest of China, has the highest prevalence of HIV among MSM in the country.

**Methods:**

Blood samples were taken from 894 MSM in Chongqing who had recently been diagnosed with HIV-1 infection and had not yet started getting treatment. In order to determine the distribution of HIV-1 subtypes, transmitted drug resistance, and assessments of molecularly transmitted clusters, we sequenced the Pol genes and employed them in phylogenetic analysis. The genetic distance between molecular clusters was 1.5%. To find potential contributing factors, logistic regression analyses were performed.

**Results:**

Of the 894 HIV-1 pol sequences acquired from study participants, we discovered that CRF07_BC (73.6%) and CRF01_AE (19.6%) were the two most prevalent HIV-1 genotypes in Chongqing among MSM, accounting for 93.2% of all infections. In addition, CRF08_BC (1.1%), B subtype (1.0%), CRF55_01B (3.4%), and URF/Other subtypes (1.3%) were less frequently observed. Among MSM in Chongqing, transmitted drug resistance (TDR) was reported to be present at a rate of 5.6%. 48 clusters with 600 (67.1%, 600/894) sequences were found by analysis of the molecular transmission network. The distributions of people by age, sexual orientation, syphilis, and genotype were significantly differentially related to being in clusters, according to the multivariable logistic regression model.

**Conclusion:**

Despite the low overall prevalence of TDR, the significance of genotypic drug resistance monitoring needs to be emphasized. CRF07_BC and CRF01_AE were the two main genotypes that created intricate molecular transmission networks. In order to prevent the expansion of molecular networks and stop the virus’s spread among MSM in Chongqing, more effective HIV intervention plans should be introduced.

## Introduction

Acquired immune deficiency syndrome (AIDS), as a serious sexually transmitted disease, is induced by the human immunodeficiency virus (HIV) and poses a threat to global population health [1]. MSM have been identified as an important population and continue to represent 23.0% of new HIV infections globally [2]. There were more than one million people living with HIV in China in 2020 [3]. The transmission of HIV among MSM has shown rapid growth in China, increasing from 2.5% of newly reported cases in 2006 to 26% in 2014 [4].

Chongqing is located in southwestern China, with a total population of over 30 million. With approximately 8000 cases reported in each of the previous three years, Chongqing is one of the most severely affected areas by HIV-1. According to Chongqing’s system of AIDS reporting, MSM made up more than 13.0% of newly diagnosed cases with HIV in this city in 2021, while more than 24.5% of cases in the downtown region (containing 9 districts) did. The high prevalence of HIV-1 among MSM was found in Southwest China. And the city of Chongqing had the greatest prevalence of HIV (13.8%, 95% CI: 12.8-14.9%) due to acceptance of homosexuality and open attitudes toward sex [5, 6]. The great mobility of the MSM population in China has been the main factor contributing to the spread of HIV among various places and groups [7, 8].

The emergence of a large range of novel circulating recombinant forms (CRFs) and unique recombinant forms (URFs) was rapid across several risk groups and regions [9]. The diversity of HIV-1 across the nation is a significant obstacle for the development of an HIV vaccine and disease prevention [10]. According to a cross-sectional national survey that included 4704 partial pol sequences, CRF01_AE (39.0%) was the most prevalent subtype of HIV-1 in 2017, followed by CRF07_BC (35.6%), CRF08_BC (8.9%), and subtype B (5.5%) [11]. CRF01_AE has had a sharp increase across almost all risk groups and geographical areas, and it has taken over among MSM in China [12]. In contrast, the earlier study found that CRF07_BC was the dominant subtype in Chongqing, China, accounting for nearly 70% of the newly diagnosed HIV infected MSM [13].

Few studies have focused on sexual behavior among MSM but have not systematically analyzed the molecular epidemiology of incident infections in Chongqing. As the HIV-1 epidemic grows among MSM, CRFs will increase and heighten the possibility of drug resistance, resulting in treatment failure [14]. Studying the epidemic of HIV-1 genetics and looking into TDR are crucial [15]. Here we use phylogenetic analyses to infer the distribution and characteristics of the subtypes of HIV-1, the TDR, and the cluster of molecularly transmitted viruses from 2018 to 2021. The results of this study could help the MSM population in Chongqing, China, with precise preventative and control strategies.

## Methods

### Study population and samples

From January 2018 to December 2021, 894 patients with HIV-1 infection were enrolled after informed consent was obtained according to the following criteria: (1) population of MSM; (2) adult residents over the age of 18 living in Chongqing; (3) The newly diagnosed HIV-infected individual and has not received treatment. Prior to the blood draw, participants’ demographic information was obtained via face-to-face interviews at two volunteer counseling and testing clinics in Jiangbei district and Yuzhong district.

### Extraction, amplification, and sequencing

According to the manufacturer’s instructions, HIV-1 RNA was isolated from a blood sample using the Shuoshi Viral RNA Kit (SDK60105, China). This HIV-1 RNA pol region encodes regions of the reverse transcriptase and protease genes (HBX2: 2147–3462), which were amplified by Reverse Transcription-Polymerase Chain Reaction (RT-PCR) technique with commercial primers according to previously published methods [16]. On a 1% agarose gel, PCR product identification was carried out using electrophoresis. The sequencing of positive products was then entrusted to Beijing Genomics Research Center Ltd. in Beijing, China.

### Sequence analysis and determination of genotypes

The software Sequencher 5.4.6 was used to modify and combine the generated sequence fragments. After that, BioEdit 7.0.9 was used to align the sequences and compare them to the reference sequence from LANL’s HIV Sequence Database. With the aid of COMET online analysis tool (https://comet.1ih.lu/index.php), genotype identification was carried out. Additionally, using the neighbor-joining approach with the Tamura-Nei 93 model in MEGA X [17], a phylogenetic tree was created. URFs were defined as sequences that could not be supported by the phylogenetic tree and COMET. We utilized the online tool ITOL (https://itol.embl.de/) to enhance the visualization of the tree and assign distinct subtype colors for labeling.

### Analysis of drug-resistance mutations

Three kind of drug classes (NRTIs, NNRTIs, and PIs) were evaluated for resistance using the online tool (https://hivdb.stanford.edu/) [18]. At least one or more TDR mutations were required for HIV-1 strains to be considered resistant.

### Construction of the transmission network

The sequences were entered into Hyphy 2.2.4 for computing the pairwise genetic distance with the Tamura-Nei 93 mode. The genetic distance between molecular clusters was 1.5% [19]. Cytoscape v3.9.0 software was then used to process and visualize the gratings.

### Statistical analysis

IBM SPSS version 22.0 program was used for all statistical analyses. We describe the qualitative statistics using frequentists. Univariate and multivariable logistic regression analyses were conducted to find potential risk factors. Statistical significance was defined as a P value < 0.05.

## Results

### Social-demographic characteristics

A total of 894 successfully amplified HIV pol sequences were obtained from 1010 research participants, with a success rate of 88.5%. These people were 31.3 years old on average (ranging from 16 to 82 years). 98.0% (876/894) were Han ethnicity, and 96.2% (860/894) were permanent residents (> 2 years). Of these subjects, 78.3% (700/894) were unmarried, 14.1% (126/894) were married or living with a spouse, and 7.6% (68/894) were divorced or widowed. The majority of the subjects (59.6%, 533/894) had a junior college or higher level of education. (Table 1)

### Sexuality-related characteristics

Among the 894 participants, 80.1% were homosexual, 15.9% were bisexual, and 4.0% were uncertain of their sexual orientation. The sex role survey showed that 34.3% (307/894) were the “Top”, 31.0% (277/894) were the “Bottom”, and 34.7% (310/894) were the “Verse”. 61.5% (550/894) had their first same-sex sex at the age of 18–24 years old. Most (81.4%, 728/894) seek sexual partners via social software. Combined rates of HIV with syphilis positivity and hepatitis C positivity were found to be 17.7% (158/894) and 0.3% (3/894) respectively.

### HIV‑1 genotypes distribution

**Fig. 1:**
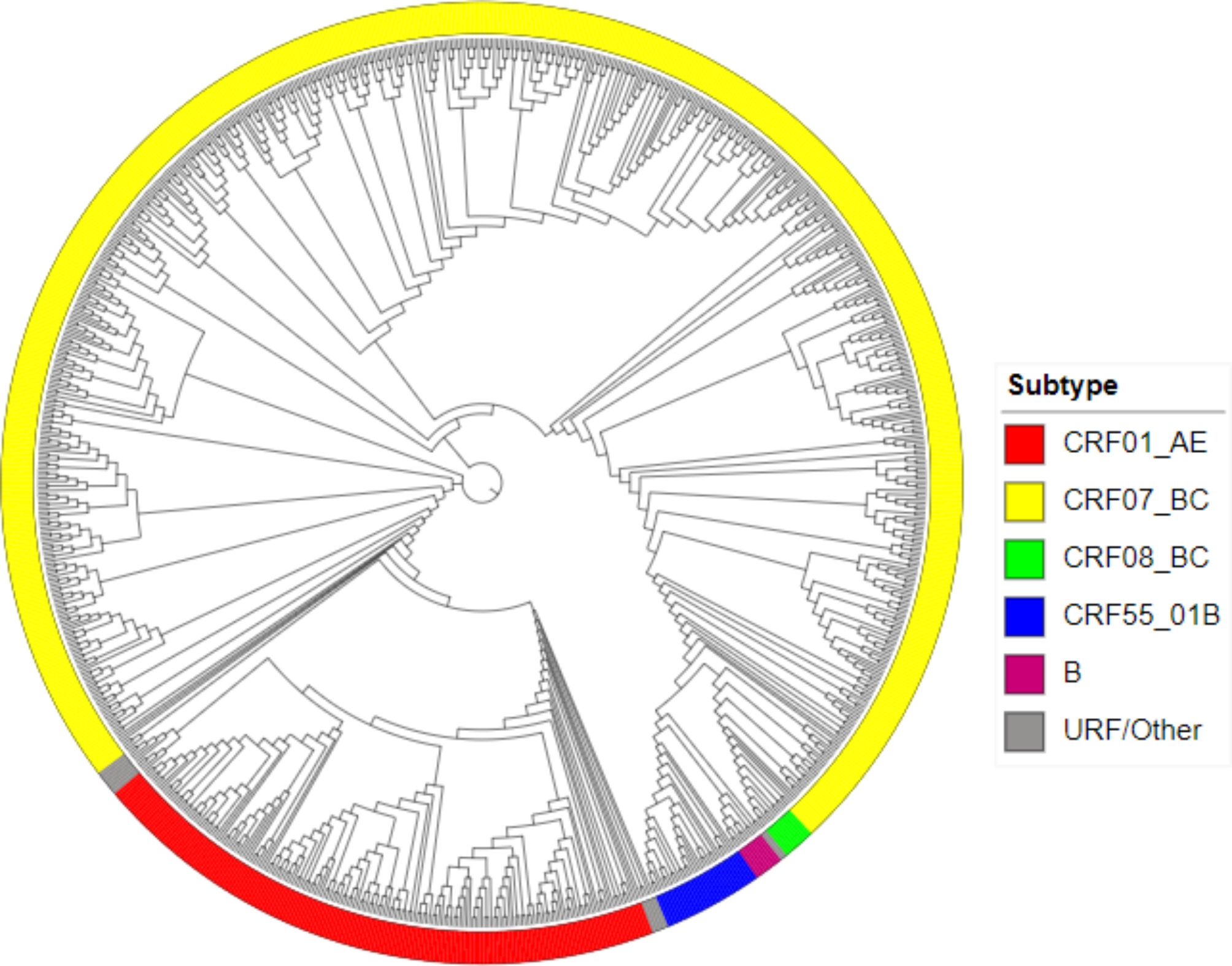
HIV‑1 genotypes distribution in the phylogenetic tree

We found that CRF07_BC (73.6%, 658/894), CRF01_AE (19.6%, 175/894) were the predominant HIV-1 genotypes circulating in Chongqing, which accounted for 93.2% of the total number of.

infections. And CRF55_01B (3.4%, 30/894), CRF08_BC (1.1%, 10/894), and subtype B (1.0%, 9/894) were less commonly observed in this study. The number of genotypes ≤ 3 (68_01B and 67_01B) and Other/URFs, were accounted for 1.3% (12/894). The color of the circle on the outside of the branch with the genotypes of CRF07_BC, CRF01_AE, CRF08_BC, CRF55_01B, B, and URF/Other shown in yellow, red, green, blue, purple, and gray, respectively, is indicated in the phylogenetic tree. (Fig. 1)

**Table 1:** Characteristics of participants

Characteristics	Number	Percent
Age (years) ≤ 24 25–34 35–44 ≥ 45	259 396 126 113	29.0 44.3 14.1 12.6
Household registrations Chongqing Other province	689 205	77.1 22.9
Local residence time ≤ 2 years > 2 years	34 860	3.8 96.2
Ethnicity Han Others	876 18	98.0 2.0
Marital status Unmarried Married or living with spouse Divorced or widowed	700 126 68	78.3 14.1 7.6
Education Illiterate or Primary Middle school High school or technical secondary school Junior college or above	32 106 223 533	3.6 11.9 24.9 59.6
Sexual orientation Homosexual Bisexual Uncertain	716 142 36	80.1 15.9 4.0
Sexual roles “Top” “Bottom” “Verse”	307 277 310	34.3 31.0 34.7
Age of first same-sex sexual behavior < 18 18–24 ≥ 25	124 550 220	13.9 61.5 24.6
Use software to find sex partners Yes No	728 166	81.4 18.6
Syphilis positive Yes No	158 736	17.7 82.3
Hepatitis C positive Yes No	3 891	0.3 99.7
Total	894	100

### Characteristics of TDR

The prevalence of TDR in MSM in Chongqing was found to be 5.6% (50/894). Of the 894 strains, Q58E (1.5%, 13/894) and M46L (0.5%, 4/894) were the most common mutations associated with PI. K103N (1.1%, 10/894), Y188L (0.6%, 5/894) and E138A (0.3%, 3/894) mutations were the most common mutations associated with NNRTI. And three strains (0.3%, 3/894) had drug resistance mutations in both PIs and NNRTIs. (Table 2)

**Table 2:** The prevalence of transmitted drug resistance mutations sites

Drug classes	Drug resistance mutations sites	Number	Percent
PI	M46I M46L M46MI G73GRS Q58E	1 4 1 1 13	0.1 0.5 0.1 0.1 1.5
NRTI	L74LI M184I M184MV T215TA V75M	1 1 1 1 1	0.1 0.1 0.1 0.1 0.1
NNRTI	A98G, K103N, V108I, E138Q, L234I E138A E138EG, V179E E138G, V179E G190S K101E K103KN K103KNRS K103N K103N, P225H V106VI, V179D V179D V179IL Y181YC Y188L	1 3 2 1 1 1 1 1 10 1 1 1 1 1 5	0.1 0.3 0.2 0.1 0.1 0.1 0.11 0.1 1.1 0.1 0.1 0.1 0.1 0.1 0.6
Total	-	50	5.6

Patients with the CRF07_BC strain (2.4%, 16/658) had the highest odds of acquiring a mutation associated with PI, followed by those with the CRF01_AE strain (2.3%, 4/175). Patients with the CRF01_AE strain (2.3%, 4/175) had the highest odds of acquiring a mutation associated with NRTI, followed by those with the CRF07_BC strain (0.2%, 1/658). The patients with the CRF55_01B and CRF08_BC strains were the most likely to acquire a mutation associated with NNRTI, followed by those who were infected with the CRF01_AE strain (5.1%, 9/175) and the CRF07_BC strain (2.7%, 18/658). Overall, the TDR prevalence rate of the CRF07_BC genotype was 7.4%, that of genotype CRF01_AE was 5.0%, that of genotype CRF08_BC was 10.0%, and that of genotype CRF55_01B was 10.0%. (Table 3)

**Table 3:** Transmitted drug resistance mutations sites in different subtypes

Subtypes	Number (%)	Number of TDR	Prevalence (%)	Prevalence (%)		
				PI	NRTI	NNRTI
CRF01_AE	175 (19.6)	13	5.0	2.3 (4/175)	2.3 (4/175)	5.1 (9/175)
CRF07_BC	658 (73.6)	33	7.43	2.4 (16/658)	0.2 (1/658)	2.7 (18/658)
CRF08_BC	10 (1.1)	1	10.0	0	0	10.0 (1/10)
CRF55_01B	30 (3.4)	3	10.0	0	0	10.0 (3/30)
B	9 (1.0)	0	0.0	0	0	0
Others or URF	12 (1.3)	0	0.0	0	0	0
Total	894 (100)	50	5.6	2.2 (20/894)	0.6 (5/894)	3.5 (31/894)

### Characteristics of molecular transmission network

**Fig. 2:**
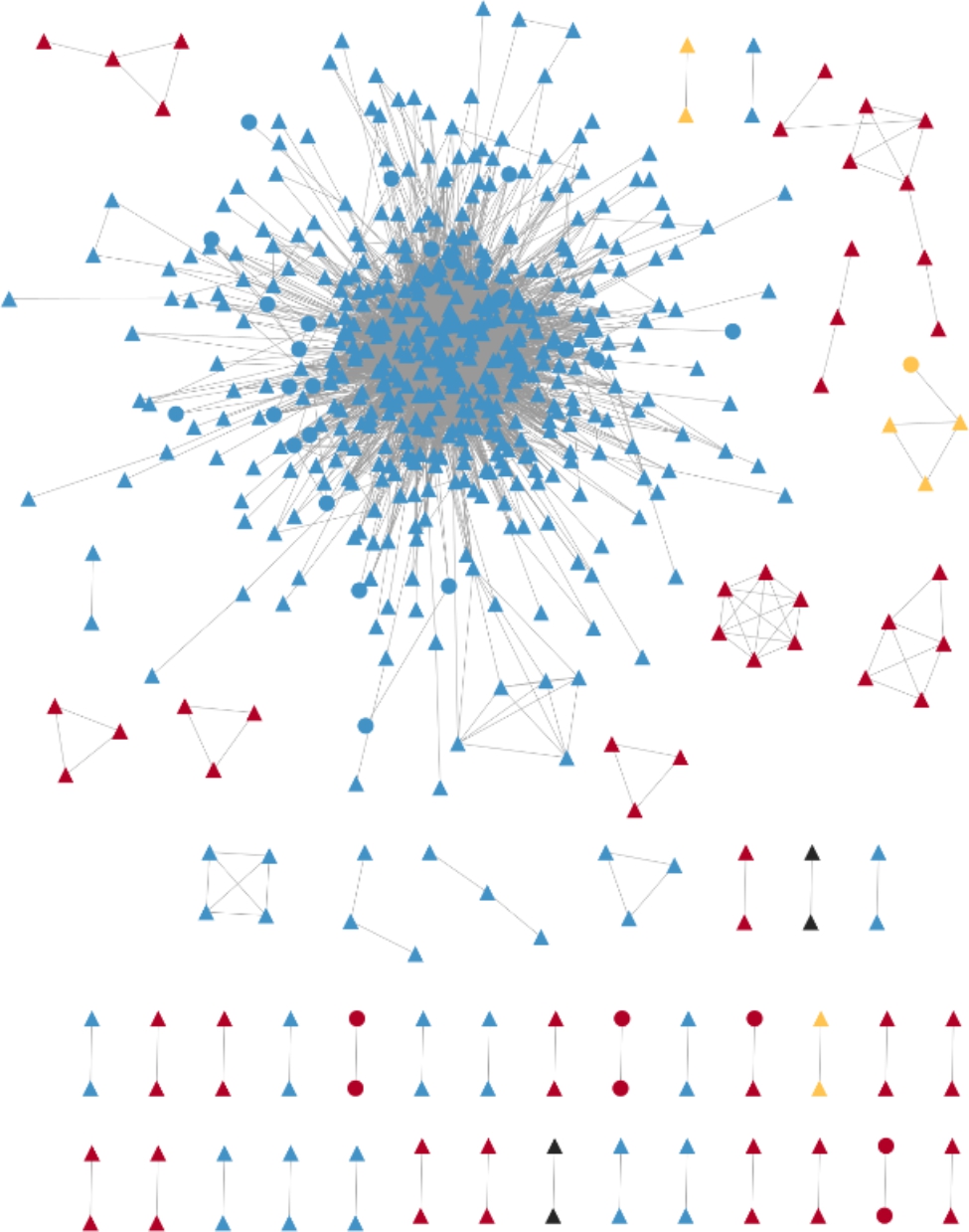
The molecular transmission network. The color of blue represents CRF07_BC, red represents CRF01_AE, orange represents CRF55_01B, and black represents others/URF; The round node indicates that there is a drug resistant mutation site, while the triangular node with no drug resistant mutation site

Analysis of the molecular transmission network identified 48 clusters containing 600 sequences (67.1%, 600/894), with 18 clusters of CRF07_BC, 25 clusters of CRF01_AE, 2 clusters of CRF55_01B, and 2 clusters of other genotypes. The largest molecular group was the CRF07_BC genotype consisting of 481 individuals and 5928 edges. (Fig. 2)

In the multivariable logistic regression model, age 23–34 years (AOR, 0.674; 95% CI 0.455–0.997) or age 35–44 years (AOR, 0.562; 95% CI 0.318–0.995), uncertain sexual orientation (AOR, 0.350; 95% CI 0.154–0.797), with no syphilis (AOR, 0.531; 95% CI 0.336–0.839), and different genotypes were significantly associated with being in clusters compared to those who were not in clusters. (Table 4)

**Table 4:** Factors associated with transmission within clusters

Characteristics		Number	In cluster	OR (95%CI)	*P*-values	AOR (95%CI)	*P*-values
Age (years)	≤ 24 25–34 35–44 ≥ 45	259 396 126 113	186 (71.2) 268 (67.7) 83 (65.9) 63 (55.8)	1 0.822 (0.583–1.158) 0.758 (0.480–1.197) 0.495 (0.312–0.783)	0.263 0.234 0.003	1 0.674 (0.455–0.997) 0.562 (0.318–0.995) 0.653 (0.314–1.536)	0.048 0.048 0.253
Household registrations	Chongqing Other province	689 205	466 (67.6) 134 (65.4)	1 0.903 (0.650–1.255)	0.544		
Local residence time	≤ 2 years > 2 years	34 860	21 (61.8) 579 (67.3)	1 1.276 (0.629–2.585)	0.499		
Ethnicity	Han Others	876 18	589 (67.2) 11 (61.1)	1 0.766 (0.294–1.996)	0.585		
Marital status	Unmarried Married or living with spouse Divorced or widowed	700 126 68	482 (68.9) 74 (58.7) 44 (64.7)	1 0.644 (0.436–0.950) 0.829 (0.492–1.398)	0.026* 0.482	1 0.897 (0.501–1.605) 1.004 (0.484–2.085)	0.713 0.991
Education	Illiterate or Primary Middle school High school Junior college or above	32 106 223 533	14 (43.8) 66 (62.3) 154 (69.1) 366 (68.7)	1 2.121 (0.952–4.727) 2.870 (1.350–6.099) 2.818 (1.369–5.801)	0.066 0.006 0.005	1 0.937 (0.355–2.472) 1.452 (0.567–3.720) 1.338 (0.528–3.389)	0.895 0.437 0.539
Sexual orientation	Homosexual Bisexual Uncertain	716 142 36	493 (68.9) 94 (66.2) 13 (36.1)	1 0.886 (0.605–1.298) 0.256 (0.127–0.514)	0.534 < 0.001	1 1.045 (0.659–1.656) 0.350 (0.154–0.797)	0.851 0.012
Sexual roles	“Top” “Bottom” “Verse	307 277 310	195 (63.5) 186 (67.1) 219 (70.6)	1 1.174 (0.834–1.652) 1.382 (0.987–1.937)	0.358 0.060		
Age of first same-sex sexual behavior	< 18 18–24 ≥ 25	124 550 220	83 (66.9) 386 (70.2) 131 (59.5)	1 1.163 (0.767–1.763) 0.727 (0.459–1.153)	0.478 0.175		
Use software to find sex partners	Yes No	728 166	499 (68.5) 101 (60.8)	1 0.713 (0.503–1.011)	0.057		
Syphilis positive	Yes No	158 736	220 (36.8) 73 (41.5)	1 0.547 (0.366–0.817)	0.003	1 0.531 (0.336–0.839)	0.007
Hepatitis C positive	Yes No	3 891	107 (40.2) 186 (36.6)	1 1.020 (0.092–11.300)	0.987		
Genotype	CRF07_BC CRF01_AE CRF08_BC CRF55_01B B Others or URF	658 175 10 30 9 12	519 (78.9) 69 (39.4) 0 (0.0) 8 (26.7) 0 (0.0) 4 (33.3)	1 0.174 (0.122–0.249) - 0.097 (0.042–0.223) - 0.134 (0.40-0.451)	< 0.001 - < 0.001 - 0.001	1 0.162 (0.112–0.234) - 0.084 (0.036–0.199) - 0.148 (0.043–0.514)	< 0.001 - < 0.001 - 0.003
Drug resistance	Yes No	50 844	35 (70.0) 565 (66.9)	1 1.152 (0.619–2.145)	0.655		

## Discussion

We performed a cross-sectional molecular epidemiologic study of HIV-1 to follow the distribution and characteristics of HIV-1 subtypes, TDR, and molecularly transmitted clusters in newly diagnosed infections in MSM in Chongqing, China. CRF07_BC was found to be the most prevalent subtype, which was in agreement with previous studies in 2012–2014 [13]. However, according to the national survey, the HIV-1 CRF01_AE strain is the most prevalent strain in China, particularly among MSM [20]. It is very odd that Chongqing has become the city with one of the highest prevalence of the CRF07_BC genotype subtype in China.

The CRF07_BC is a variant of an HIV-1 B’ and C recombination in persons who inject drugs (PWID) in southwestern China during the 1990s, it also spread to other cities and groups of heterosexuals at first via the drug trafficking route [21]. Then, because of the aggressive fight against drug trafficking and the introduction of methadone maintenance treatment programs [22], CRF07_BC has increasingly spread through sexual contact among MSM [5, 23]. Thus, we hypothesize that HIV flowed into Xinjiang and Yunnan Province through the transmission of drug use in the early years and then spread to become the dominant strain in the Chongqing region. There were also a few studies that suggested that CRF07_BC strains are commonly detected in MSM in certain regions of China, such as Jilin [24] and Shijiazhuang[25].

In our study, in addition to CRF07_BC, CRF01_AE also represented 19.6% of all participants which was less than that of the survey conducted in Shanghai [26] and Tianjin [27]. CRF01_AE transmitted primarily through the sexual route spread from the southeastern coast and southwestern border to the entire country [28]. It is also noteworthy that the CRF55_01B strain represents a higher percentage than CRF08_BC and the B subtype, despite being a relatively “young” HIV strain in MSM. CRF55_01B was first reported in Changsha, Hunan province, and Dongguan, Guangdong Province, China in 2013 [29] and has since spread rapidly across the country [30]. In a recent study, CRF55_01B, despite originating from MSM, was found to be transmitted among heterosexuals, indicating that heterosexual males played a critical role in the transmission and dissemination of this strain [31].

This study suggests that DRMs influencing the efficacy of NNRTIs, followed by NRTIs and PIs that were in line with other cities in China [11]. TDF, 3TC, and EFV are the most frequently utilized drugs in China’s current first-line therapy guidelines [32]. First-line treatment medications can still be used constantly in Chongqing because the aforementioned three drug kinds demonstrated that resistance was largely at a low potential level. K103N observed as the most frequent mutation in response to NNRTIs was reported in Anhui [33] and Guangdong [34] provinces and is also consistent with the results of our study.

HIV molecular transmission network can help us formulate precise interventions and improve the efficiency of public health [35]. For the purposes of this study, we identify all clusters with the genetic distance of ≤ 1.5%, which has been shown to be more appropriate for molecular networks of more than three years [36]. Within these transmission clusters, we found an enormous CRF07_BC cluster comprising 422 sequences suggestive of a strong association in MSM in Chongqing. Due to this complex transmission relationship, once the dominant strain has been identified, it will still continue, which to some degree, accounts for the consistently high proportion of CRF07_BC within Chongqing.

We discovered that being in a cluster was highly correlated with age, sexual orientation, syphilis, and genotypes. Age is one such factor, which is best recognized because MSM in their younger years engage in greater sexual activity and have more complicated molecular transmission networks [37]. Syphilis is a sexually transmitted illness that increases the chance of contracting HIV and, to some extent, reflects the state of the sexual activity network [38].

The MSM who self-identified as homosexual engaged in sexual conduct more frequently than those who were less convinced of their sexual orientation [39]. As a result, different sexual orientations displayed varied rates of network entry. The two main genotype transmission clusters in the network, CRF07_BC and CRF01_AE, need to be dynamically monitored in order to allow for precise preventive and control actions to stop their transmission.

## Conclusion

Despite the low overall prevalence of TDR, the significance of genotypic drug resistance monitoring needs to be emphasized. CRF07_BC and CRF01_AE were the two main genotypes that created intricate molecular transmission networks. In order to prevent the expansion of molecular networks and stop the virus’s spread among MSM in Chongqing, more effective HIV intervention plans should be introduced.

## Data Availability

The corresponding author can provide all of the datasets used in this study upon reasonable request.

## Abbreviations

AIDS: Acquired immunodeficiency syndrome
HIV: Human immunodeficiency viruses
MSM: Men who have sex with men
STDs: Sexually transmitted diseases
SDRMs: Surveillance/transmitted drug resistance mutations
TDR: Transmitted drug resistance
TN93: Tamura-Nei 93
URFs: Unique recombinant forms
ART: Antiretroviral therapy
CRFs: Circulating recombinant forms
NNRTIs: Nonnucleoside reverse transcriptase inhibitors
NRTIs: Nucleoside reverse transcriptase inhibitors
PIs: Protease inhibitors
URFs: Unique recombinant forms
